# Molecular Epidemiology of Extraintestinal Pathogenic *Escherichia coli* Causing Hemorrhagic Pneumonia in Mink in Northern China

**DOI:** 10.3389/fcimb.2021.781068

**Published:** 2021-10-28

**Authors:** Ying Yu, Bo Hu, Huanhuan Fan, Hailing Zhang, Shizhen Lian, Hongye Li, Shuangshuang Li, Xijun Yan, Shaohui Wang, Xue Bai

**Affiliations:** ^1^ Key Laboratory of Special Animal Epidemic Disease, Ministry of Agriculture, Institute of Special Animal and Plant Sciences, Chinese Academy of Agricultural Sciences, Changchun, China; ^2^ Shanghai Veterinary Research Institute, Chinese Academy of Agricultural Sciences, Shanghai, China

**Keywords:** *Escherichia coli*, hemorrhagic pneumonia, serotype, virulence, multilocus sequence typing

## Abstract

The molecular epidemiology and biological characteristics of *Escherichia coli* associated with hemorrhagic pneumonia (HP) mink from five Chinese Provinces were determined. From 2017 to 2019, 85 *E. coli* strains were identified from 115 lung samples of mink suffering from HP. These samples were subjected to serotyping, antimicrobial susceptibility, detection of virulence genes, phylogenetic grouping, whole-genome sequencing, drug resistant gene, multilocus sequence typing (MLST) and biofilm-forming assays. *E. coli* strains were divided into 18 serotypes. Thirty-nine *E. coli* strains belonged to the O11 serotype. Eighty-five *E. coli* strains were classified into seven phylogenetic groups: E (45.9%, 39/85), A (27.1%, 23/85), B1 (14.1%, 12/85), B2 (3.7%, 3/85), D (3.7%, 3/85), F (2.4%, 2/85) and clade I (1.2%, 1/85). MLST showed that the main sequence types (STs) were ST457 (27/66), All *E. coli* strains had ≥4 virulence genes. The prevalence of virulence was 98.8% for *yijp* and *fimC*, 96.5% for *iucD*, 95.3% for *ompA*, 91.8% for *cnf-Ⅰ*, 89.4% for *mat*, 82.3% for *hlyF*, and 81.2% for *ibeB.* The prevalence of virulence genes *iss*, *cva/cvi*, *aatA*, *ibeA*, *vat*, *hlyF*, and *STa* was 3.5–57.6%. All *E. coli* strains were sensitive to sulfamethoxazole, but high resistance was shown to tetracycline (76.5%), chloramphenicol (71.8%), ciprofloxacin (63.5%) and florfenicol (52.9%), resistance to other antibiotics was 35.3–16.5%. The types and ratios of drug-resistance genes were *tet(A), strA, strB, sul2, oqxA, blaTEM-1B, floR, and catA1* had the highest frequency from 34%-65%, which were consistent with our drug resistance phenotype tetracycline, florfenicol, quinolones, chloramphenicol, the *bla-NDM-I* and *mcr-I* were presented in ST457 strains. Out of 85 *E. coli* strains, six (7.1%) possessed a strong ability, 12 (14.1%) possessed a moderate ability, and 64 (75.3%) showed a weak ability to form biofilm. Our data will aid understanding of the epidemiological background and provide a clinical basis for HP treatment in mink caused by *E. coli*.

## Introduction

*Escherichia coli* is a Gram-negative opportunistic pathogen. It is a normal flora of the gastrointestinal tract of humans and warm-blooded animals (Abdollah et al., 2018). However, if the immune system is compromised, *E. coli* can cause various intestinal and extraintestinal diseases.

Pathogenic *E. coli* can be divided into enteropathogenic and extraintestinal types. Extraintestinal pathogenic *E. coli* can cause colisepticemia, urinary-tract infections, and meningitis (Devine et al., 1989; Tibbetts et al., 2003; Gaschignard et al., 2011; Derakhshandeh et al., 2018). Tibbetts and colleagues isolated 40 *E. coli* strains from the livers, spleens, and lungs of mink (*Neogale* species) suffering from colisepticemia (Tibbetts et al., 2003). In that study, *E. coli* was shown, for the first time, to be associated with hemorrhagic pneumonia (HP) in mink. In China, Zhang and colleagues isolated *E. coli* strains from the lungs of mink suffering from respiratory symptoms (Zhang et al., 2019). That study provided additional evidence of the correlation between *E. coli* and HP in mink.

HP in mink is almost exclusively seasonal (from September to early December) and is characterized by sudden death (Qi et al., 2014). HP caused by *Pseudomonas aeruginosa* was described in Denmark in 1953 (Knox, 1953). In 1985, Han and colleagues were the first to report HP outbreaks in mink each year in China (Han et al., 2014). *Klebsiella pneumoniae* is another pathogen causing HP in mink. Fifteen *K. pneumoniae* strains were isolated from mink experiencing respiratory distress in China, and most of isolates were identified as serotype K2 (Wang et al., 2017). In that study, the mink showed lung hemorrhage, liver hemorrhage/swelling, slight bleeding in the brain, and liver abscess. The histology lesions of mink with acute HP were found to be associated with infection with *P. aeruginosa* and *E. coli* in a study by Hammer et al. (2013). They discovered that *P. aeruginosa* was most often found surrounding blood vessels and in the alveolar lining, whereas *E. coli* showed a more diffuse distribution in lung tissue. Although the pathogenesis is different, HP in mink caused by *E. coli* merits attention, but little information is available.

We investigated the disease characteristics, serotyping, phylogenetic groups, multilocus sequence typing (MLST), whole-genome sequencing, drug-susceptibility, and detection of virulence factors on *E. coli* strains. Our study will aid understanding of the epidemiological background of HP and provide a clinical basis for its treatment in mink caused by *E. coli*.

## Materials and Methods

### Collection of Samples

**Figure 1:**
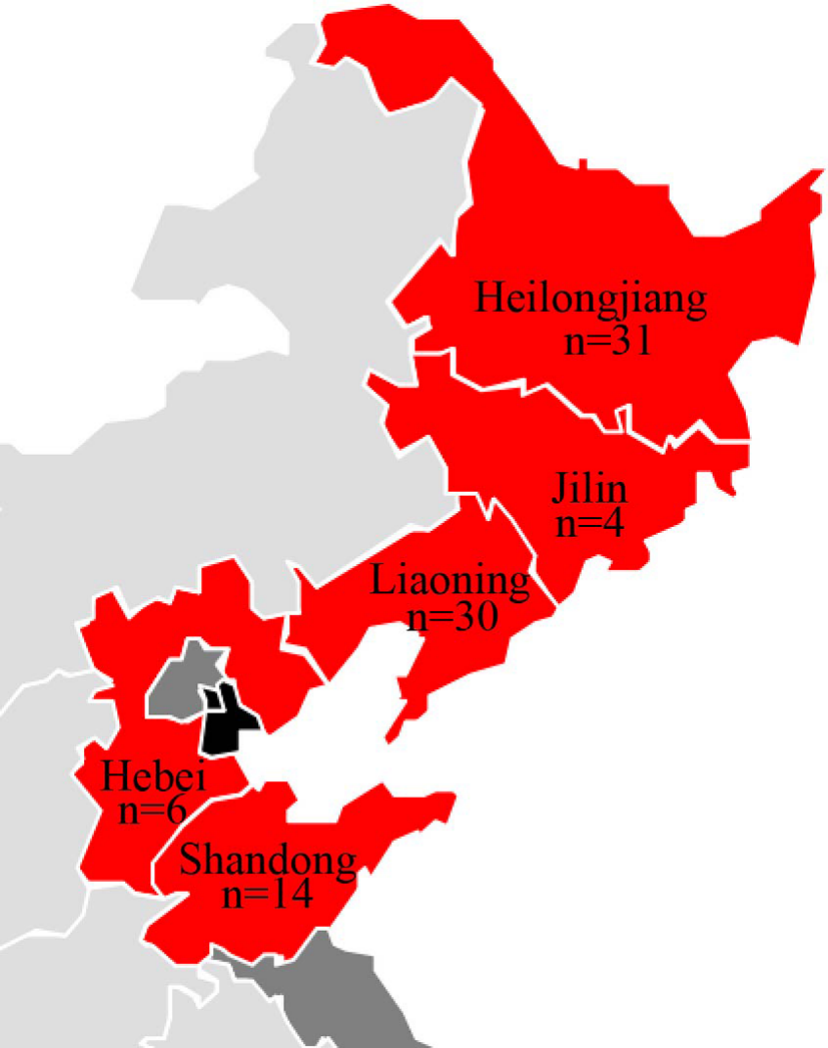
Geographic distribution of E. coli isolates from minks in this study.

Between March 2017 and February 2019, 115 minks who died of HP were collected from 21 farms in northern China (Jilin, Liaoning, Heilongjiang, Hebei, and Shandong Provinces) (**Figure 1**). The minks showed clinical pneumonia signs, including: high fever (>40°C), severe depression and anorexia, dyspnea, coughing, as well as hematemesis of the mouth and nose.The dead minks underwent dissection, and lung samples were used for bacterial isolation.

### Histopathology

After necropsy, samples were fixed in 10% neutral-buffered formalin. Then, they were embedded in paraffin wax and stained with hematoxylin and eosin for histopathology following standard procedures (Bancroft and Stevens, 1990).

### Isolation and Identification of *E. coli*

Isolation and identification of *E. coli* strains were undertaken according to standard procedures with some modifications (Qiu et al., 2019). Lung samples were inoculated onto MacConkey agar. Bacterial colonies were selected and cultured in Luria–Bertani (LB) broth at 37°C. These bacterial isolates were identified by classical biochemical methods and confirmed by 16S rRNA-sequencing. All *E. coli* strains were grown in LB broth at 37°C with aeration and stored at −80°C in 20% glycerol for further use.

### DNA Extraction

The genomic DNA of *E. coli* isolates was extracted using the TIANamp Bacteria DNA Kit according to manufacturer's (Tiangen, Beijing, China) instructions.

### Serotyping

Traditional agglutination test and O-genotyping PCR were used to identify the O serotypes of the *E. coli* isolates. Briefly, serotyping of *E. coli* isolates was done by agglutination test with specific serum against *E. coli* O antigens (Statens Serum Institut, Copenhagen, Denmark) according to the manufacturer’s guidelines. The O-genotyping PCR were carried out through the implication of O antigen biosynthesis genes as described previously (Iguchi et al., 2015).

### Antimicrobial Susceptibility Testing

The minimal inhibitory concentration of 85 *E. coli* strains was determined according to the Performance Standards for Antimicrobial Susceptibility Testing of the Clinical Laboratory Standards Institute (CLSI; M100-S23). Each strain was evaluated for susceptibility to the cefotaxime, tetracycline, chloramphenicol, florfenicol, levofloxacin, ciprofloxacin, sulfamethoxazole, colistin, gentamicin, and spectinomycin. The susceptibility of *E. coli* isolates was interpreted referring to CLSI M100-S23. *E. coli* ATCC25922 were used as a quality-control strain in antimicrobial susceptibility testing.

### Phylogenetic Group Analysis

*E. coli* strains were classified as eight phylogenetic groups using the rapid phylogenetic grouping PCR as described previously. Thus, the phylogenetic groups of *E. coli* isolates were determined *via* PCR detection of genes *chuA, yjaA, TspE4.C2*, *arpA*, and *trpA* according to Clermont protocol (Clermont et al., 2013) (**Table 1**).

**Table 1:** Primers employed for detection of phylogenetic groups.

PCR type	Primer ID	Target	Primer sequence (5′-3′)	PCR product (bp)
Quadruplex	chuA.1b	chuA	ATGGTACCGGACGAACCAAC	288
	chuA.2		TGCCGCCAGTACCAAAGACA	
	yjaA.1b	yjaA	CAAACGTGAAGTGTCAGGAG	211
	yjaA.2b		AATGCGTTCCTCAACCTGTG	
	TspE4C2.1b	TspE4.C2	CACTATTCGTAAGGTCATCC	152
	TspE4C2.2b		AGTTTATCGCTGCGGGTCGC	
	AceK.f	arpA	AACGCTATTCGCCAGCTTGC	400
	ArpA1.r		TCTCCCCATACCGTACGCTA	
Group E	ArpAgpE.f	arpA	GATTCCATCTTGTCAAAATATGCC	301
	ArpAgpE.r		GAAAAGAAAAAGAATTCCCAAGAG	
Group C	trpAgpC.1	trpA	AGTTTTATGCCCAGTGCGAG	219
	trpAgpC.2		TCTGCGCCGGTCACGCCC	
Internal control	trpBA.f	trpA	CGGCGATAAAGACATCTTCAC	489
	trpBA.r		GCAACGCGGCCTGGCGGAAG	

### Distribution of Virulent Genes

All strains were tested for the presence of 24 virulence-associated genes by simplex PCR and multiplex PCR (Tibbetts et al., 2003; Meng et al., 2014): *LT, STa, STb, SLT-Ⅰ*, *cnf-Ⅰ*, *cnf-Ⅱ, eae, K99, aatA, papC, tsh*, *fimC, mat, ibeB, vat, yijp, ibeA*, *ompA, neuC*, *cva/cvi*, *iss*, *fyuA*, *iucD* and *hlyF*. The primers used for detection are listed in **Table 2**. PCRs were conducted under identical reaction conditions according to those described previously (Tibbetts et al., 2003; Meng et al., 2014).The amplification products were visualized by agarose gel (1%) electrophoresis.

**Table 2:** Primers used for detection of virulent genes.

Primer	Sequence (5′-3′)	Product size/bp	Reference
*LT-F*	TATCCTCTCTATATGCACAG	480	Tibbetts et al., 2003
*LT-R*	CTGTAGTGGAAGCTGTTATA		
*STa-F*	GCCTATGCATCTACACAATC	244	Tibbetts et al., 2003
*STa-R*	ATAACATCCAGCACAGGCAG		
*STb-F*	GCTATGCATCTACACAATC	278	Tibbetts et al., 2003
*STb-R*	TGAGAAATGGACAATGTCCG		
*SLT-F*	ACACTGGATGATCTCAGTGG	600	Tibbetts et al., 2003
*SLT-R*	CTGAATCCCCCTCCATTATG		
*cnf-I-F*	GAACTTATTAAGGATAGT	543	Tibbetts et al., 2003
*cnf-I-R*	CATTATTTATAACGCTG		
*cnf-II-F*	AATCTAATTAAAGAGAAC	543	Tibbetts et al., 2003
*cnf-II-R*	CATGCTTTGTATATCTA		
*eae-F*	GTGGCGAATACTGGCGAGAC	890	Tibbetts et al., 2003
*eae-R*	CCCCATTCTTTTTCACCGTCG		
*K99-F*	TGGGACTACCAATGCTTCTG	450	Tibbetts et al., 2003
*K99-R*	TATCCACCATTAGACGGAGC		
*aatA-F*	CATAGGCGTTTCTCTTTCCGAT	1226	Meng et al., 2014
*aatA-R*	CCTGTCGTTCATACAGATTCGTT		
*papC-F*	GCTGATATCACGCAGTCAGT	768	Meng et al., 2014
*papC-R*	GTCAACAAGAAGACGTGTTCC		
*tsh-F*	GTCTGTCAGACGTCTGTGTTTC	598	Meng et al., 2014
*tsh-R*	ATAGGATGACAGGCTACCGAC		
*fimC-F*	GCCGATGGTGTAAAGGATGG	475	Meng et al., 2014
*fimC-R*	GGGTAAGTGCGCCATAATCA		
*mat-F*	CGACCTGGTCAGCAACAGCC	238	Meng et al., 2014
*mat-R*	TCCACGCCCACATTCAGTGT		
*ibeB-F*	GTTCTCACTCAGCCAGAACG	1172	Meng et al., 2014
*ibeB-R*	CATCCAGCACTTCCAGATAAC		
*vat-F*	TCCATGCTTCAACGTCTCAGAG	939	Meng et al., 2014
*vat-R*	CTGTTGTCAGTGTCGTGAACG		
*yijp-F*	TGGCTTGATTCTGCATCCGAT	517	Meng et al., 2014
*yijp-R*	CATCGTCTGCTGGTTGGTGAT		
*ibeA-F*	GTATGACGGTGGGAACAAGAG	321	Meng et al., 2014
*ibeA-R*	TGGCAATAGCAGCGGCAGTC		
*ompA-F*	AGCTATCGCGATTGCAGTG	919	Meng et al., 2014
*ompA-R*	GGTGTTGCCAGTAACCGG		
*neuC-F*	GGTGGTACATTCCGGGATGTC	792	Meng et al., 2014
*neuC-R*	CATGGTGGTGAAAAGACATTAGC		
*cva/cvi-F*	TCCAAGCGGACCCCTTATAG	598	Meng et al., 2014
*cva/cvi-R*	CGCAGCATAGTTCCATGCT		
*iss-F*	ATCACATAGGATTCTGCCG	309	Meng et al., 2014
*iss-R*	CAGCGGAGTATAGATGCCA		
*fyuA-F*	ACACGGTTTATCCTCTGGC	953	Meng et al., 2014
*fyuA-R*	GGCATATTGACGATTAACGAA		
*iucD-F*	ACAAAAAGTTCTATCGCTTCC	714	Meng et al., 2014
*iucD-R*	CCTGATCCAGATGATGCTC		
*hlyF-F*	GGCCACAGTCGTTTAGGGTGCTTACC	450	Meng et al., 2014
*hlyF-R*	GGCGGTTTAGGCATTCCGATACTCAG		

### Whole-Genome Sequencing

Whole-genome sequencing (WGS) was used to characterize the resistome, MLST and evaluate genetic evolution in 66 representative strains. The whole genome was sequenced by Beijing Novogene Bioinformatics Technology (Beijing, China). The sequence of genomic DNA in cells was identified (PacBio Single Molecule, Real-Time Sequencing; Pacific Biosciences, Menlo Park, CA, USA). The neighbor-joining method (>5 samples) was employed to construct an evolutionary tree using TreeBeST Vision 1.9.2. Resistance genes were predicted by comparing the sequenced strains with a database on antiviral resistance genes (http://katholt.github.io/srst2/).

### MLST

Sixty-six *E. coli* strains were underwent MLST by comparing of seven housekeeping genes (*adk*, *fumC*, *gyrB*, *icd*, *mdh*, *purA*, and *recA*) according to WGS results and aligned against the allele templates of *E. coli* retrieved from an online database (http://www.mlst.net/). A minimum spanning tree and clustering tree were generated using BioNumerics 7.6 (www.applied-maths.com/download/software/).

### Quantification of Biofilm Formation

Biofilm-formation assays were undertaken, as described previously (Vogeleer et al., 2015) with some modifications. Briefly, *E. coli* strains were cultured in LB overnight at 37°C and diluted into LB at 1:100. A 200-μL aliquot of each dilution was dispensed into individual wells of a sterile 96-well microtiter plate and incubated for 36 h at 37°C. Wells with sterile LB medium served as negative controls. Then, the culture medium was discarded, and the wells were washed thrice with phosphate-buffered saline. After fixation with methanol for 15 min, biofilms were stained with 0.1% crystal violet for 5 min. The plates were washed five times to remove unbound dye and air-dried. Finally, the biofilms were quantified by measuring the optical density (OD) at 595 nm with a microplate reader(eppendorf, BioPhotomete) after dissolving in 33% glacial acetic acid for 30 min. The capability to form a biofilm was scored in accordance with criteria described previously (Stepanovic et al., 2000): OD< ODc, non-producer; ODc < OD < 2×ODc, weak producer; 2× ODc < OD < 4 × ODc, moderate producer; OD >4ODc, strong producer. “OD” denoted the OD at 595 nm of *E. coli*, whereas “ODc” denoted three standard deviations above the mean OD of the negative control. Tests were carried out thrice, and the results were averaged.

## Results

### Organ Symptoms and Hispathological Changes

**Figure 2:**
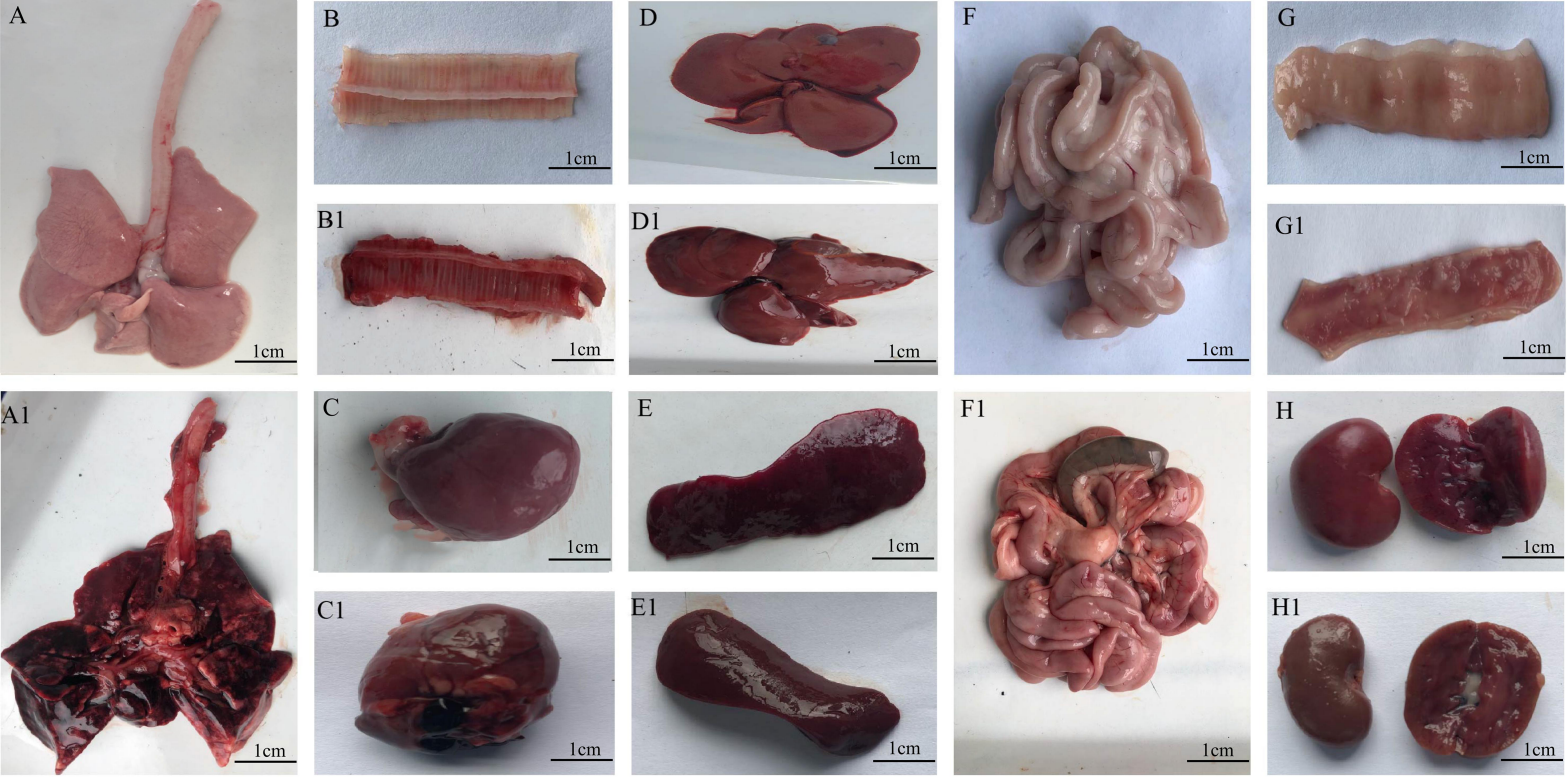
Organs from minks after *E. coli* infection. **(A1–G1)** Organs of infected minks. **(A–G)** Organs of heathy minks. **(A, A1)** Lung. **(B, B1)** trachea. **(C, C1)** Heart. **(D, D1)** Liver. **(E, E1)**. Spleen. **(F, F1)** Inner wall of the intestinal tract. **(G, G1)** Kidney. **(H, H1)** Renal hemorrhage.

The onsets of minks were breathing difficulties, blood-like exudates around the nostrils/mouths, and, in some cases, foaming around the mouth, The most common observation were edematous, extensive hemorrhage and pleural effusion. hyperemia or hemorrhagic spots and ecchymosis were observed in lung. Other symptoms were liver hemorrhage, renal hemorrhage, spleen enlargement, and black bleeding spots. The intestinal surface and inner wall were dark-red or pink, which indicated a small amount of bleeding. Swelling and bleeding of inguinal, submandibular, and mesenteric lymph nodes were also documented (**Figure 2**).

**Figure 3:**
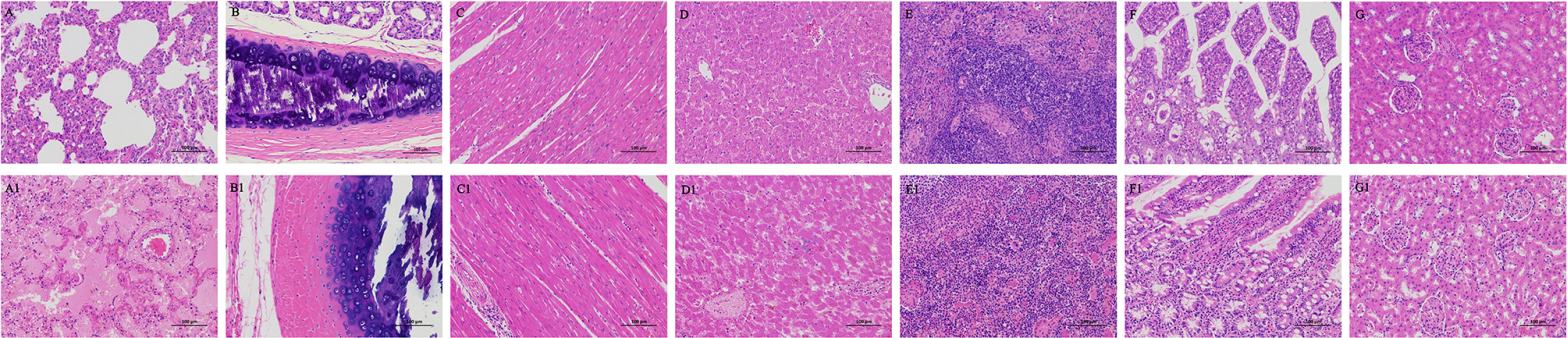
Hispathological changes of Organs from minks after *E. coli* infection. **(A–G)** Organs of infected minks. **(A1–G1)** Organs of heathy minks. **(A, A1)** Lung. **(B, B1)** trachea. **(C, C1)** Heart. **(D, D1)** Liver. **(E, E1)**. Spleen. **(F, F1)** the intestinal tract. **(G, G1)** Kidney.

The hispathological changes of organs were as follows, lung, severe pulmonary edema, a large number of eosinophilic serous exudation in alveolar cavity; tracheae, cell necrosis, nuclear fragmentation or dissolution in mucosal layer; heart and liver: lymphocytic infiltration around blood vessels; spleen, massive extramedullary hematopoietic foci and multinucleated giant cells; Intestine, no obvious inflammation; kidney, necrotic shedding of renal tubular epithelial cells (**Figure 3**).

### Isolation and Identification of *E*. *coli* Strains

A total of 115 lung samples from 21 mink farms were cultured. Eighty-five *E. coli* isolates were identified by conventional biochemical methods. 16S rRNA-sequencing indicated that these strains had features consistent with *E. coli*.

### Serotyping

**Figure 4:**
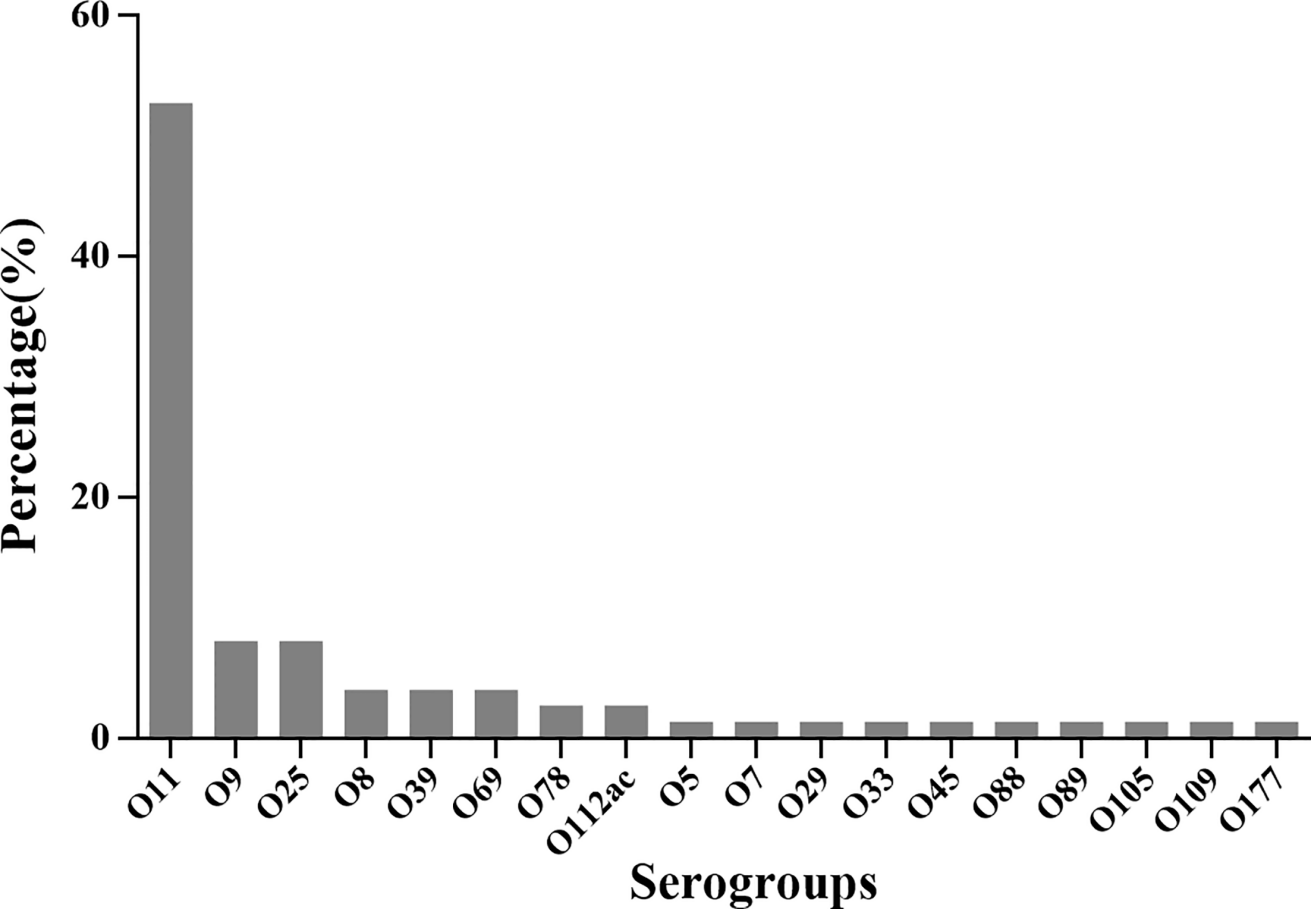
The serotype distribution of 85 isolated *E. coli* strains.

Serotyping showed that 87.1% (74/85) of *E. coli* isolates could be classified into the single-O type, whereas 11 isolates could not be classified into any serotype. Of these serotyped isolates, 18 O serotypes were identified (**Figure 4**). The most prevalent serotype were O11 (39/85), followed by O9 (6/85), O25 (6/85), O8 (3/85), O39(3/85), O69(3/85), O78(2/85), and O112ac (2/85). Only one strain belonged to O5, O7, O29, O33, O45, O88, O89, O105, O109, and O177 serotypes, respectively. These results indicated that O11, O9, and O25 were the predominant serotypes of extraintestinal pathogenic *E. coli* from mink in a particular population in northern China.

### Antimicrobial Susceptibility Testing

**Figure 5:**
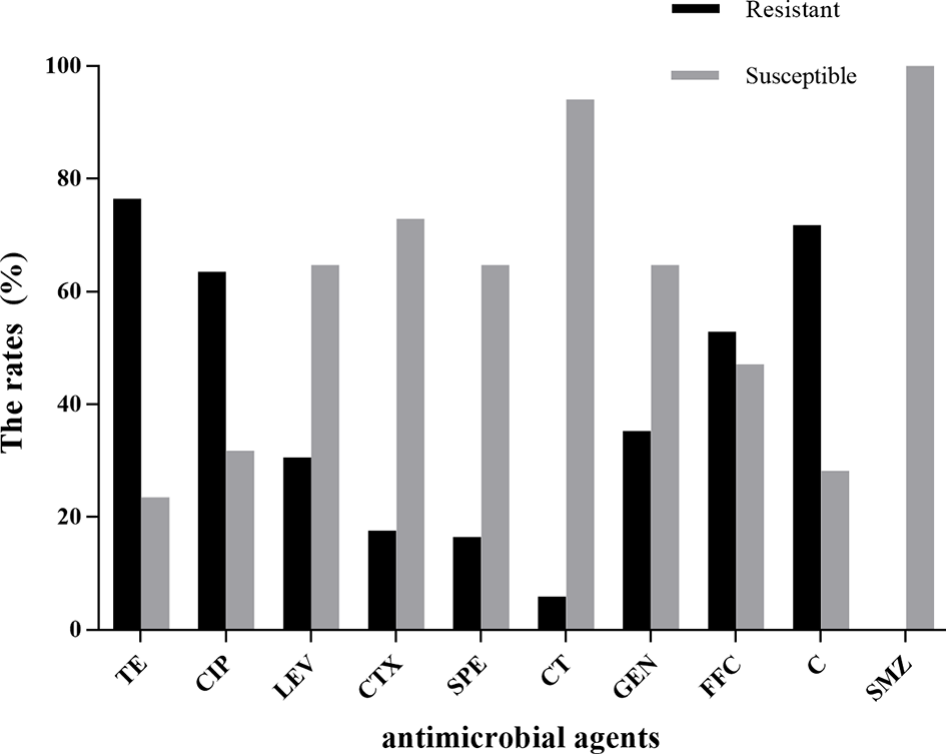
Results of antimicrobial susceptibility testing of 85 *E. coli* strains. TE, tetracycline; CIP, ciprofloxacin; LEV, levofloxacin; C, chloramphenicol; GEN, gentamicin; FFC, florfenicol; CTX, cefotaxime; SPE, spectinomycin; CT, colistin; SMZ, sulfamethoxazole.

The results of the antimicrobial susceptibility test are shown in **Table S1 of Supplementary Materials**. Eighty-five *E. coli* strains were sensitive to sulfamethoxazole, but they showed high resistance to tetracycline (76.5%), chloramphenicol (71.8%), ciprofloxacin (63.5%), and florfenicol (52.9%). Resistance to colistin was noted in only 5.9% of *E. coli* strains. Resistance to the other antibiotics tested was 16.5%–35.3% (**Figure 5**). We analyzed the susceptibility of *E. coli* strains to 10 antibiotics, and found that 55 of 85 (64.7%) strains were resistant to ≥3 antibiotics.

### Distribution of Virulence Genes

**Figure 6:**
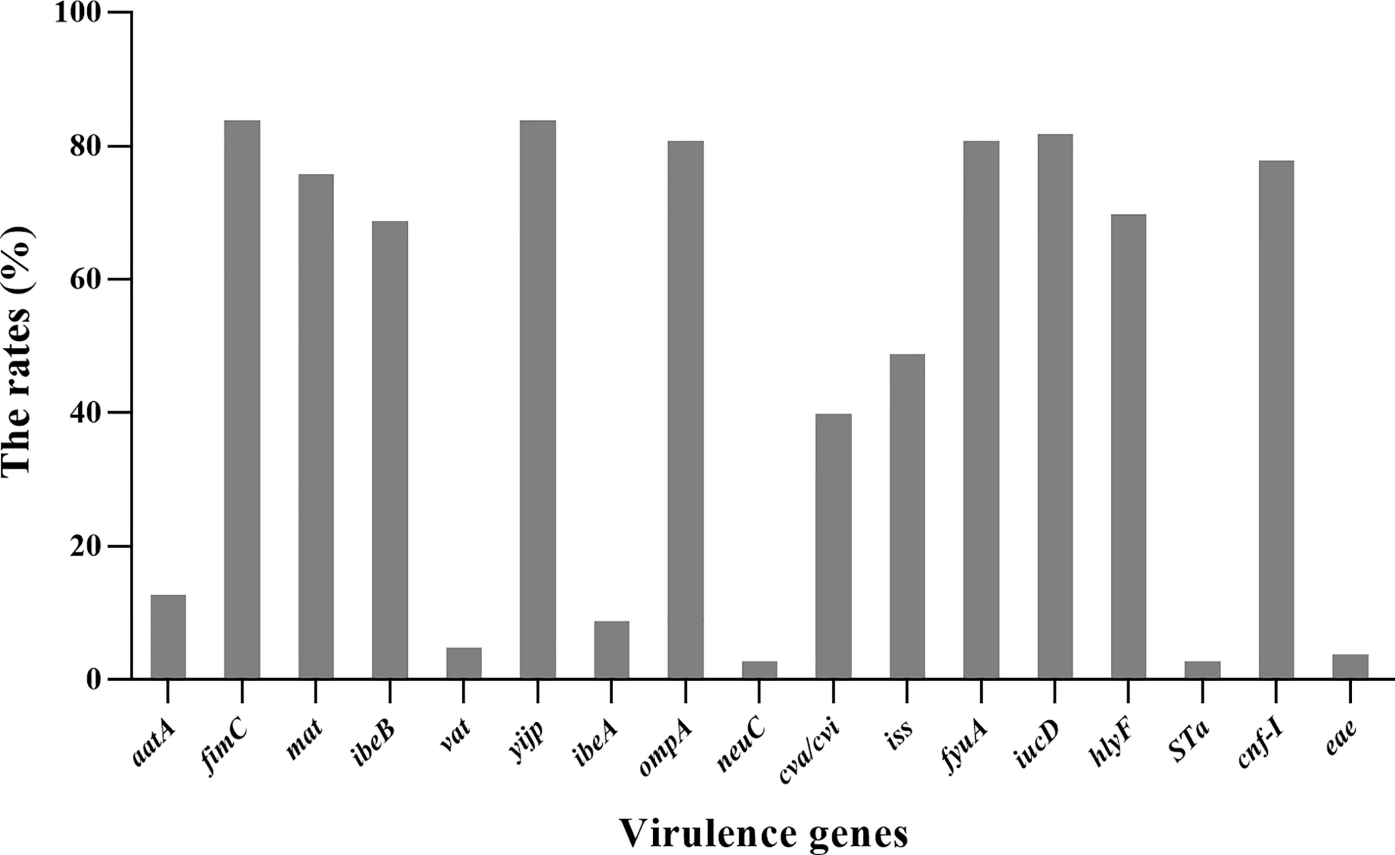
Distribution of virulence genes in 85 *E. coli* strains.

All 85 *E. coli* strains were screened for the presence of 24 virulence genes. *yijp* and *fimiC* were detected in 84 (98.8%) strains. *iucD*, *ompA*, *cnf-Ⅰ*, and *fyuA* were detected in 82 (96.5%), 81 (95.3%), 81 (95.3%), and 78 (91.8%) strains, respectively. *mat*, *hlyF*, and *ibeB* were present in 76 (89.4%), 70 (82.3%), and 69 (81.2%) of strains, respectively. *iss* and *cav/cvi* were detected in 49 (57.6%) and 40 (47.1%) of strains, respectively. *aatA*, *ibeA*, *vat*, and *eae* were detected in 13 (15.3%), 9 (10.6%), 5 (5.9%), and 4 (4.7%) strains, respectively. Moreover, three (3.7%) strains harbored *STa* and *nueC*. In general, all *E. coli* strains contained ≥4 virulence genes. However, *LT*, *STb*, *SLT-Ⅰ*, cnf*-Ⅱ*, *K99*, *papC*, and *tsh* were not detected (**Figure 6**).

### Phylogenetic Grouping

**Figure 7:**
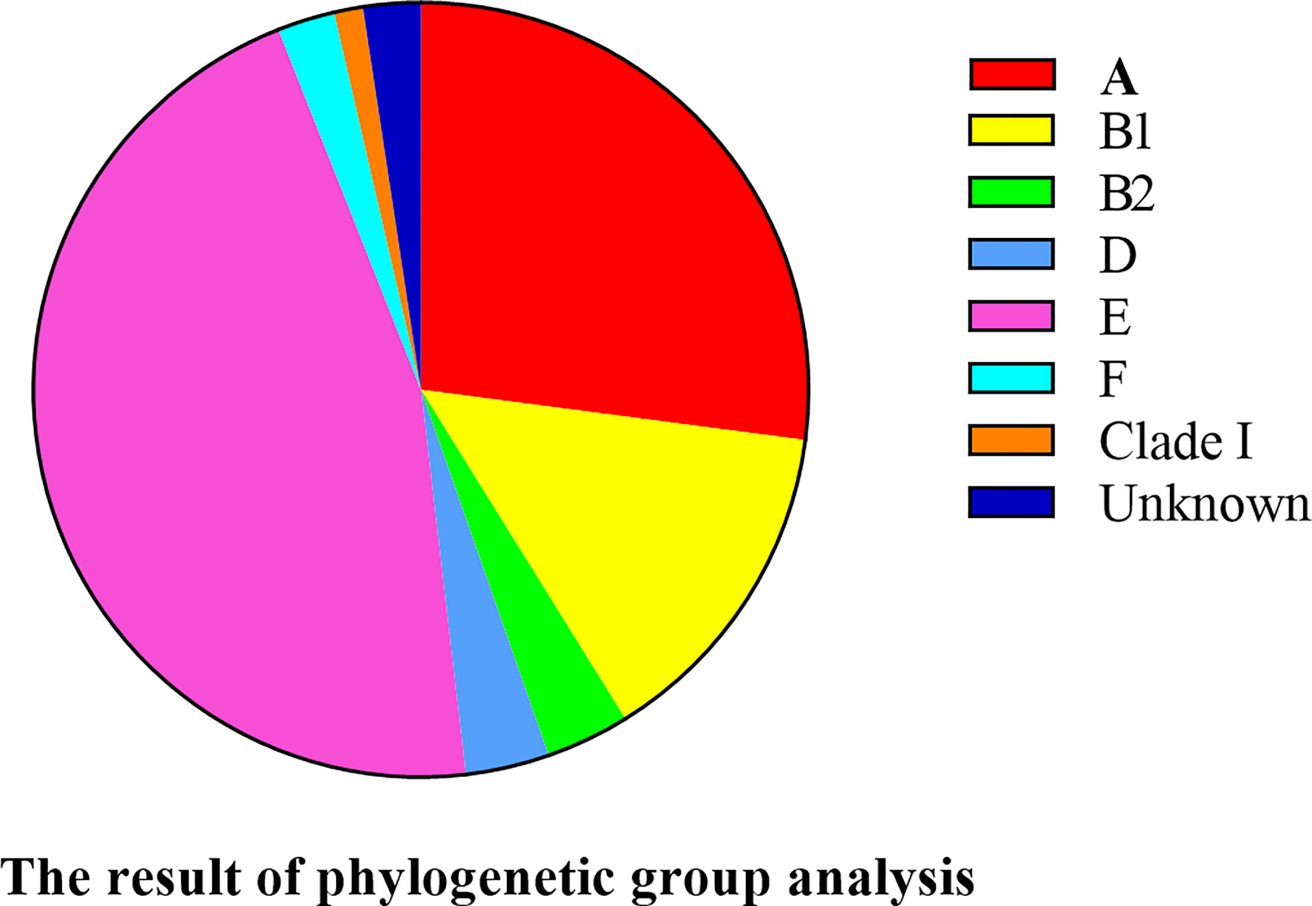
Phylogenetic classification of 85 *E. coli* strains.

Phylogenetic typing showed that most of *E. coli* strains belonged to group E (45.9%, 39/85) and A (27.1%, 23/85). Only 3.5% of *E. coli* strains belonged to groups B2 and D, 2.4% to F, and 1.2% to clade I. However, two *E. coli* strains were not classified into any phylogenetic group (**Figure 7**). Thus, the *E. coli* strains that caused HP in mink were strongly associated with phylogenetic groups A and E.

Most virulence genes were associated with phylogenetic groups E and A. *yijp*, *fimC*, *iucD*, *ompA*, and *fyuA* showed a wide distribution among all groups (A, B1, B2, D, E, F, and clade I). *cnf-Ⅰ*, *hlyF*, *iss*, and *cva/cvi* were less prevalent in clade I. *vat* was associated with group B2 and clade I. *STa* was found only in groups A and E. *nueC* was associated only with groups A and group B2 (**Table 3**).

**Table 3:** Relationship among phylogenetic groups and virulence genes.

Gene	Phylogenetic group (n, %)							Total
	A (n =23)	B1 (n = 12)	B2 (n = 3)	D (n = 3)	E (n = 39)	F (n = 2)	Clade Ⅰ (n = 1)	
*fimC*	23 (100%)	12 (100%)	3 (100%)	3 (100%)	39 (100%)	2 (100%)	1 (100%)	83
*yijp*	23 (100%)	12 (100%)	3 (100%)	3 (100%)	38 (97.4%)	2 (100%)	1 (100%)	82
*iucD*	23 (100%)	12 (100%)	3 (100%)	3 (100%)	37 (94.9%)	2 (100%)	1 (100%)	81
*fyuA*	23 (100%)	12 (100%)	3 (100%)	3 (100%)	37(94.9%)	1 (50%)	1 (100%)	80
*ompA*	21 (91.3%)	12 (100%)	3 (100%)	3 (100%)	38 (97.4%)	2 (100%)	1 (100%)	80
*cnf-Ⅰ*	18 (78.3%)	12 (100%)	3 (100%)	3 (100%)	37 (94.9%)	2 (100%)	0	74
*mat*	20 (87%)	8 (66.7%)	3 (100%)	2 (66.7%)	38 (97.4%)	2 (100%)	1 (100%)	74
*hlyF*	17 (73.9%)	9 (75%)	3 (100%)	2 (66.7%)	36 (92.3%)	2 (100%)	0	69
*ibeB*	18 (78.3%)	6 (50%)	3 (100%)	2 (66.7%)	36 (92.3%)	2 (100%)	1 (100%)	68
*iss*	14 (66.7%)	7 (58.3%)	3 (100%)	2 (66.7%)	21 (53.8%)	2 (100%)	0	49
*cva/cvi*	4 (17.4%)	4 (33.3%)	2 (66.7%)	1 (33.3%)	26 (66.7%)	2 (100%)	0	39
*aatA*	6 (26.1%)	1 (8.3%)	0	1 (33.3%)	5 (12.8%)	0	0	13
*ibeA*	0	2 (16.7%)	0	1 (33.3%)	6 (15.4%)	0	0	9
*vat*	0	0	3 (100%)	0	0	0	1 (100%)	4
*eae*	1 (4.3%)	2 (16.7%)	0	0	1 (2.6%)	0	0	4
*STa*	2 (8.7%)	0	0	0	2 (5.1%)	0	0	3
*nueC*	2 (8.7%)	0	1 (33.3%)	0	0	0	0	3

### Whole-Genome Sequencing

**Figure 8:**
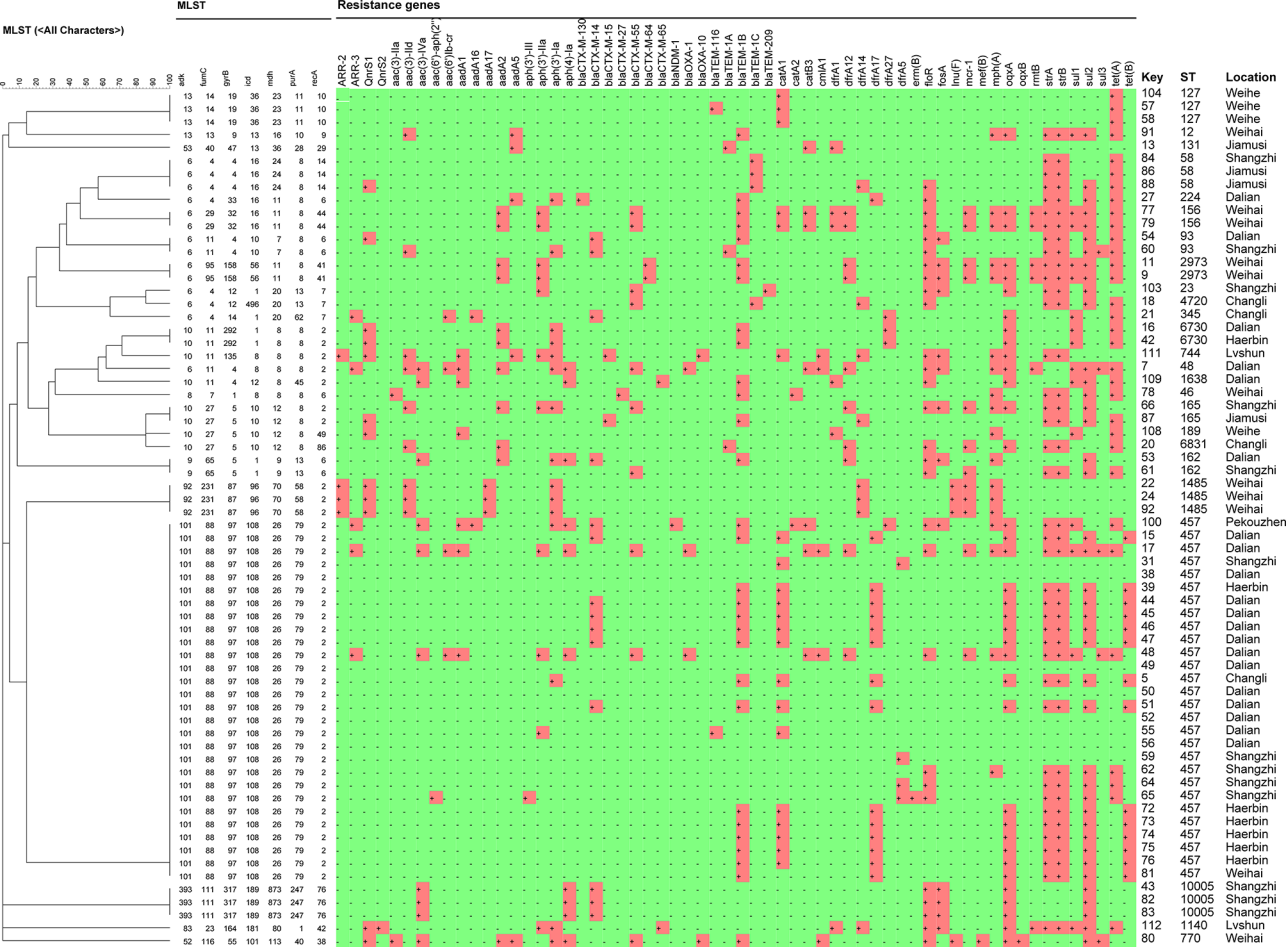
Clustering tree of 66 *E. coli* strains using multilocus sequence typing by Whole-genome sequencing analysis. The red cube region represents the presence of a drug-resistance gene.

Whole-genome sequencing (WGS) was done on the genome data of the new strains in the present study. The complete genome MLST clustering tree showed that the *E. coli* strains from mink were far from the reference strains. The *E. coli* strains in mink showed regional aggregation and transmission. The homology of the *E. coli* strains from Weihai, Dalian, Shangzhi, Harbin, and Weihe reached 100%, respectively. However, the other strains were far from each other (**Figure 8**).

**Figure 9:**
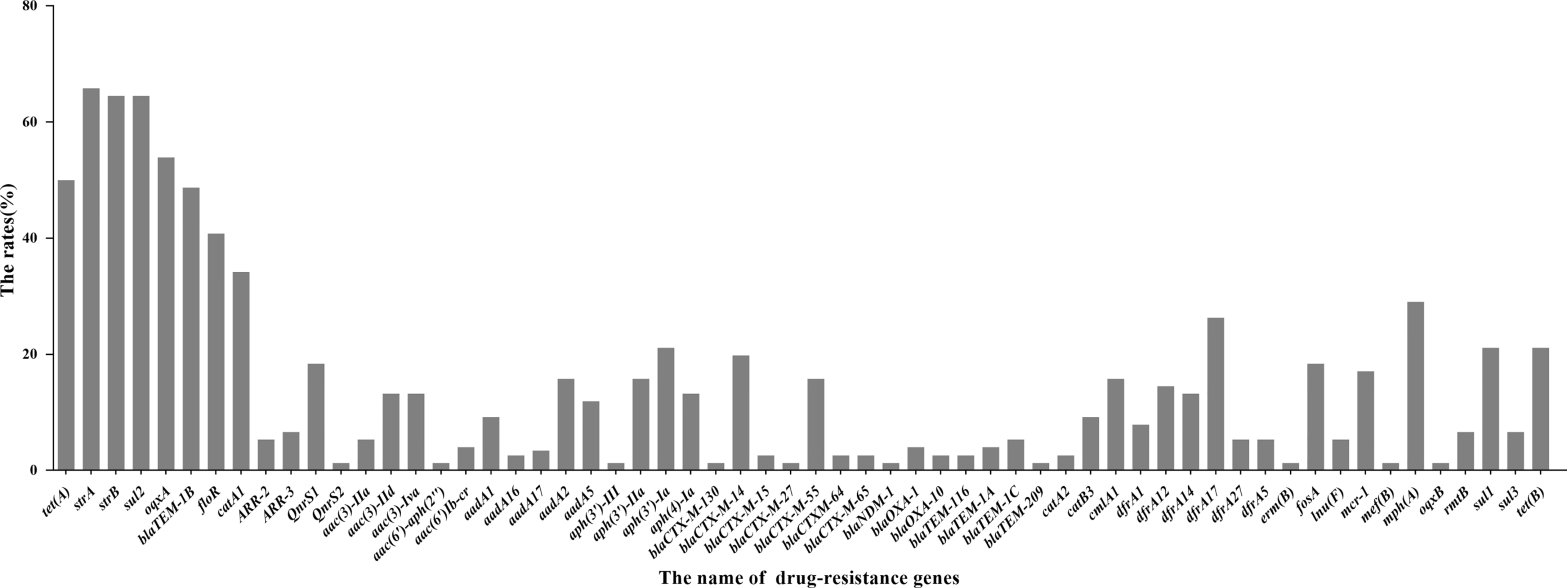
Distribution of drug-resistant genes of 66 *E. coli* strains by Whole-genome sequencing analysis.

We predicted the types and ratios of drug-resistance genes in 66 strains of *E. coli* by whole-gene sequencing. *tet(A), strA, strB, sul2, oqxA, blaTEM-1B, floR, and catA1* had the highest frequency at 50%, 65.8%, 64.5%, 64.5%, 53.9%, 48.7%, 40.8%, and 34.2%, respectively. The other drug-resistance genes were rangely from 1.3%∼29%, (**Figure 9**). the highest frequency antibiotic genes (ARGs) were resistant to tetracycline, florfenicol, quinolones, chloramphenicol, which were consistent with our drug resistance phenotype.

### MLST

**Figure 10:**
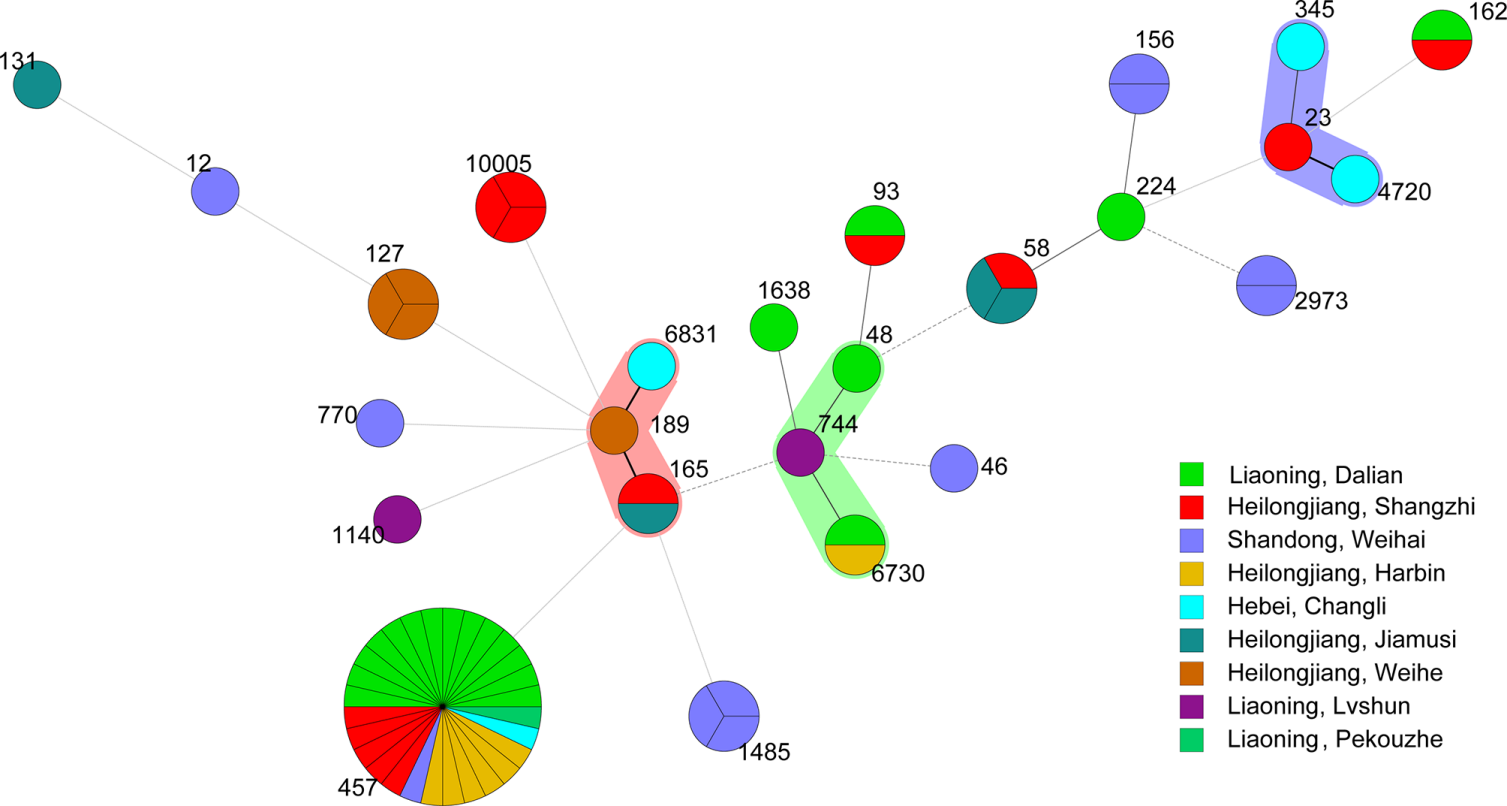
Minimum spanning tree of 66 *E. coli* strains by Whole-genome sequencing analysis. Each circle represents one ST. The area of the circle corresponds to the number of strains. The color of the circle indicates the area to which the strain belongs.

Sixty-six *E. coli* strains were classified into 28 sequence types (STs). The dominant types were ST457 (twenty-seven strains from six outbreaks), followed by ST127 (three strains from one outbreak), ST1485 (three strains from one outbreak), ST10005 (three strains from one outbreak), ST58 (two strains from two outbreaks), ST6730 (two strains from two outbreaks), ST93 (two strains from two outbreaks), ST2973 (two strains from two outbreaks), ST162 (two strains from two outbreaks), and ST156 (two strains from one outbreak). The only one strain of ST was ST23, ST58, ST189, ST1638, ST744, ST1140, ST131, ST165, ST4720, ST6831, ST345, ST224, ST48, ST46, ST770, ST7, ST5, and ST12 (**Figure 10**).

### Quantification of Biofilm Formation

Out of 85 *E. coli* strains, six (7.1%) possessed strong biofilm-forming ability, 12 (14.1%) possessed moderate biofilm-forming ability, and 64 (75.3%) showed weak biofilm-forming ability. Three *E. coli* strains could not form biofilms (**Table S1 in Supplementary Materials**).

## Discussion

HP in mink was first discovered in China in 1983 (Han et al., 2014). *P. aeruginosa* and *K. pneumoniae* are the important agents of HP; they are the major causes of death and induce economic loss in the mink industry (Wang et al., 2017, Zhao et al., 2018).

We isolated 85 *E. coli* strains from 115 minks with HP. We assessed the serotype, phylogenetic group, and virulence genes of these *E. coli* strains. We undertook, whole-genome sequencing, MLST, antimicrobial susceptibility testing and quantification of biofilms to provide a theoretical basis for the prevention and treatment of HP in mink.

O11 was the predominant (45.9%) serotype of the 85 *E. coli* strains in the lung samples of mink with HP. Serotype O11 has been reported to be the most prevalent among isolates from pigs with postweaning diarrhea and birds with colibacillosis (Chen et al., 2004; Hussein et al., 2013). Our data are consistent with findings from Xu and colleagues showing that two out of five *E. coli* strains belonged to serotype O11 (Xu et al., 2018). The other serotypes we detected, including O9, O25, and O8, have been described to be most frequently related to urinary-tract infections, bacteremia, and diarrhea (Devine et al., 1989; Tibbetts et al., 2003; Wani et al., 2004). The serotypes of *E. coli* are not found commonly in other types of disease in mink (Tibbetts et al., 2003). This disparity may be the result of genetic variations and regional differences.

The distribution and frequencies of serotypes varied considerably from region to region (**Table S1 in Supplementary Material**). Serotype O11 was distributed in four Provinces (Liaoning, Heilongjiang, Shandong, Jilin and Hebei). The other dominant serotypes (O8, O9 and O25) were in Shandong Province. Serotypes O39, O69, O78, O112ac, O5, O7, O29, O33, O45, O88, O89, O105, O109, and O177 were less common.

Phylogenetic analyses have shown that 95% of *E. coli* strains fall into eight phylogenetic groups: A, B1, B2, C, D, E, F, and clade I (Clermont et al., 2013; Silva et al., 2017). Primary infection with *E. coli* leading to persistence or relapse of infection is associated with phylogenetic group B2, whereas primary infection with *E. coli* followed by cure or reinfection is associated with phylogenetic group D (Ejrns, 2011), respectively. We also found that *E. coli* strains isolated from infected mink belonged mainly to phylogenetic groups A and E, and other *E. coli* strains belonged to phylogenetic groups B1, B2, D, F, and clade I. This distribution of *E. coli* strains is different to that reported in bovine mastitis and broiler chickens (Hussein et al., 2013; Zhang et al., 2017), but is consistent with the distribution of human enteroinvasive *E. coli* (Da Silva et al., 2017). Our results provide helpful references of the ecological distribution and genetic evolution of *E. coli* in mink in northern China.

Virulence genes have important roles in pathogenic *E. coli*. Hence, we detected the distribution of extraintestinal virulence gene markers in *E. coli* strains from mink (Tenglin et al., 2018). The virulence genes mainly encoded genes related to invasion, toxins, adhesion, antiserum survival, and iron transport. *yijp, fimC*, *iucD*, *ompA*, *cnf-Ⅰ*, *mat, hlyF*, and *ibeB* had a high presence (98.8%, 98.8%, 96.5%, 95.3%, 91.8%, 89.4%, 82.3% and 81.2%, respectively) but some had prevalence <60% (*iss*, *cva/cvi*, *aatA*, *ibeA*, *vat*, and *eae*). The resluts proofed that *E.coli* strains isolated in this study were extra-intestinal strains. the profiles of virulent genes were the same as other extra-intestinal strains, Tibbetts found that 47.5% of the test strains had *cnf-I* gene,and 7.5% of the strains had *eae* gene in mink with colisepticaemia (Tibbetts et al., 2003). Paixão found that the frequency of avian *E.coli* virulence gene *fimC*, *iucD*, *cva/cvi*, *iss* was 92.91%, 65.35%, 55.12% and 33.07% (Paixão et al., 2016).

MLST is a bacterial-typing method based on determination of the sequence of nucleic acids. MLST has been used for epidemiological monitoring and evolutionary studies. In our study, 28 STs were found. The results are different from *E. coli* strains isolated from mink feces in Zhucheng (Qiu et al., 2019), These differences may suggest that, compared with *E. coli* isolated from mink feces, the genetic information and pathogenicity of E. coli isolated from mink lungs are different. In our study, the dominant type was ST457(27/66, 40.9%). which has the potential cause antimicrobial-resistant extraintestinal infection, almost certainly in dogs, wild animals and possibly in humans (Nicolas-Chanoine et al., 2014). ST457 in *E. coli* isolated from dog feces contains *bla-CMY* and *bla-CTX-M-15*, which are resistant to fluoroquinolone (Guo et al., 2015), ST457 in *E. coli* isolated from blood contains *blaCTX-M-1* and mcr*-1* (Kuo et al., 2016), ST457 in *E. coli* isolated from bovine mammary glands is associated with resistance to aminoglycoside and trimethoprim, respectively (Suzuki et al., 2016). The clustering tree showed that the resistance genes of ST457 in *E. coli* were similar (**Figure 8**). ST127 can cause intestinal infections in humans, dogs, and cats (Johnson et al., 2008), and it is a biological model of *E. coli* infection outside the intestine (Hochhut et al., 2006). ST1485 and ST131 in *E. coli* are related to human infection. ST131 in *E. coli* is an extraintestinal pathogen. Pitout and colleagues showed that invasion by ST131 is closely related to colonization, iron-absorption capacity, and the ability to form biofilms (Pitoust and Devinney, 2017).

According to the cgMLST clustering tree, HP outbreak in mink had the characteristics of regional aggregation and transmission. We suggest that farmers should pay attention to detection and isolation during the start of an HP outbreak in mink. We found that the main drug-resistance genes were *tet(a), sul2, oqxa, flor, blatem-1b, cata1, stra*, and *strB*, Profiles for antibiotic resistance and resistance genes for ST genotypes with different genetic relationships were documented. The distribution of drug-resistance genes was similar in the same ST type, as shown in **Figure 5**.

Use of antimicrobial drugs can lead to resistance to their effects (Mackenzie et al., 2014; Wang et al., 2017). A vaccine to prevent extraintestinal pathogenic *E. coli* in mink is not available. We analyzed the susceptibility of *E. coli* strains to 10 antibiotics. All *E. coli* strains were susceptible to sulfamethoxazole but showed high resistance to tetracycline (76.5%), chloramphenicol (71.8%), ciprofloxacin (63.5%), and florfenicol (52.9%). Resistance to multiple drugs was observed, with 55 of 85 (65.7%) isolates resistant to ≥3 antibiotics. Pedersen and colleagues also showed *E. coli* to be susceptible to sulfamethoxazole and resistant to tetracycline (54.7%) and spectinomycin (18.5%) (Pedersen et al., 2009). However, those findings were not in accordance with those of Tibbetts and coworkers, who reported that the *E. coli* strains from mink with colisepticemia were sensitive to ciprofloxacin but resistant to sulfamethoxazole (63%), gentamycin (20%), and chloramphenicol (8%) (Tibbetts et al., 2003). The reasons for these inconsistencies may be the different sources and regional differences of *E. coli* strains.

A biofilm is composed of surface-bound or sessile microbes enclosed in an amorphous extracellular matrix (Donlan and Costerton, 2002). Residence in a biofilm community offers bacteria an enhanced ability to cause disease (Wirth et al., 2006; Wang et al., 2011). We found that 75.3% of *E. coli* strains had a weak ability to form a biofilm. This weakness is obviously different for strains isolated from other species; toxin-producing strains usually possess a strong ability to form biofilms (Vogeleer et al., 2015). *E. coli* strains isolated from poultry often possess a moderate ability to form a biofilm (Wang et al., 2016). We found that strains that could form a strong biofilm were resistant to tetracycline, ciprofloxacin, and chloramphenicol.

### Conclusions

The *E. coli* strains isolated from mink were extraintestinal pathogenic and the dominant serotype was O11. A and E were the predominant phylogenetic groups among *E. coli* strains in the parts of northern China we sampled. *E. coli* isolated from mink lungs had similar genes, and some strains had the characteristics of regional aggregation and transmission. Although these strains were resistant to multiple drugs, sulfonamides are first-line treatment for HP caused by *E. coli* in mink in China. Through the study of extraintestinal pathogenic *E. coli* in mink, we aim to develop a triple vaccine using *P. aeruginosa*, *K. pneumoniae*, and extraintestinal pathogenic *E. coli* against HP in mink.

## Data Availability Statement

The data presented in the study are deposited in the NCBI repository, accession number listed in the **Supplementary Materials Table S1**.

## Ethics Statement

The animal study was reviewed and approved by the Ethics Committee of Institute of Special Animal and Plant Sciences, Chinese Academy of Agricultural Sciences. Written informed consent was obtained from the owners for the participation of their animals in this study.

## Author Contributions

YY, BH, and HZ conducted the experiments. SW and XB designed the experiments. YY and HF analyzed the data. SZL and HL assisted with the experiments. YY wrote the manuscript. SW and XB revised the manuscript. SW and XB have contributed equally to this work. All authors contributed to the article and approved the submitted version.

## Funding

This work was funded by the Science and Technology Development Project of Jilin Province (20200402111NC).

## Conflict of Interest

The authors declare that the research was conducted in the absence of any commercial or financial relationships that could be construed as a potential conflict of interest.

## Publisher’s Note

All claims expressed in this article are solely those of the authors and do not necessarily represent those of their affiliated organizations, or those of the publisher, the editors and the reviewers. Any product that may be evaluated in this article, or claim that may be made by its manufacturer, is not guaranteed or endorsed by the publisher.
